# Impact of Fish Meal Replacement with Meat Meal on the Growth and Fillet Nutritional Quality of Chum Salmon (*Oncorhynchus keta*)

**DOI:** 10.3390/foods15193475

**Published:** 2026-09-28

**Authors:** Seong-Mok Jeong, Jinho Bae, Min-Gi Kim, June Kim, Daehyun Ko, Hye Jeong Park, Tae Sook Na, Mi-Soon Jang

**Affiliations:** Aquafeed Research Center, National Institute of Fisheries Science, Buk-gu, Pohang 37517, Republic of Korea; jinhobae@korea.kr (J.B.); kmg0415@korea.kr (M.-G.K.); rlawns6766@naver.com (J.K.); iiiilliill@naver.com (D.K.); meorange44@gmail.com (H.J.P.); gidtn9568@naver.com (T.S.N.)

**Keywords:** *Oncorhynchus keta*, fish meal replacement, meat meal, n-3 LC-PUFA, hepatic gene expression, fatty acid biosynthesis

## Abstract

Rendered animal by-products such as meat meal are promising fish meal alternatives owing to their high protein content; however, they contain little n-3 long-chain polyunsaturated fatty acids (LC-PUFA), whose effects on fillet fatty acid quality and endogenous LC-PUFA biosynthesis in chum salmon (*Oncorhynchus keta*) remain unclear. We evaluated the growth, fillet fatty acid composition, and hepatic gene expression of chum salmon fed four diets—a control with 50% fish meal (D1), diets with 35% or 20% fish meal and the remainder replaced by meat meal (D2 and D3), and a D3-like diet with reduced fish oil (D4). Growth performance and feed utilization were unaffected, whereas the hepatosomatic index decreased as meat meal inclusion increased. Muscle n-3 LC-PUFA decreased with fish meal replacement but did not differ between D3 and D4. Serum non-specific immune parameters were unaffected, whereas serum GPx and catalase activities were lowest in fish fed D3. Although *srebf1* (sterol regulatory element-binding transcription factor 1) expression increased at the highest replacement level, *fads2* (fatty acid desaturase 2) and *elovl5* (elongation of very long-chain fatty acids protein 5) were not upregulated, indicating that the reduced n-3 LC-PUFA supply was not compensated by endogenous biosynthesis. Overall, meat meal can replace up to 60% of fish meal without compromising growth, but adequate dietary n-3 LC-PUFA should be maintained to preserve fillet quality.

## 1. Introduction

Fish meal is a high-quality protein source with high digestibility and is rich in essential amino acids and n-3 long-chain polyunsaturated fatty acids (LC-PUFA), making it the most widely used protein source in aquaculture feed [1]. However, limited global supply has driven higher fish meal prices, increased costs, and sustainability concerns, making reduced use essential for sustainable aquaculture growth [2,3]. Researchers have explored alternative protein sources, including plant-derived ingredients and animal by-product meals [4,5,6].

Chum salmon (*Oncorhynchus keta*) is a cold-water fish species distributed throughout the North Pacific region, including the Bering Sea, Alaska, and East Asia [7,8]. Over the past decades, the return rate of wild fish has declined, prompting increased interest in the aquaculture industry for this species [9,10,11]. However, most studies exploring fish meal replacements for salmonids have focused on Atlantic salmon (*Salmo salar*) and rainbow trout (*Oncorhynchus mykiss*), so species-specific feed formulations for chum salmon remain scarce [12,13,14].

Among available fish meal replacement options, plant proteins are widely used because of their stable availability, relatively low cost, and sustainability; however, antinutritional factors, amino acid imbalances, and reduced palatability often constrain their use [15]. In contrast, rendered animal by-products such as meat meal offer a promising, cost-effective alternative to fish meal. Meat meal is produced from underutilized parts in the meat processing industry and provides a high crude protein content with a favorable amino acid profile. Utilizing these co-products in aquafeed not only valorizes agricultural waste but also reduces the industry’s reliance on marine resources, which explains the growing research interest in its application [16,17]. Nonetheless, meat meal has relatively high ash and phosphorus concentrations, low taurine content, and a fatty acid profile dominated by saturated and monounsaturated fatty acids with minimal n-3 LC-PUFA. Therefore, replacing fish meal with meat meal would alter the dietary amino acid and taurine supply and markedly lower the dietary n-3 LC-PUFA available for deposition in fish tissues.

Atlantic salmon possess fatty acid desaturases (Fads), which catalyze the introduction of double bonds into fatty acid chains, and elongases of very long-chain fatty acids (Elovl), which catalyze carbon-chain extension, indicating a limited capacity to synthesize LC-PUFAs from dietary precursors [18]. However, this capacity varies among species and dietary contexts, and it remains unclear whether chum salmon can upregulate this pathway to compensate for n-3 LC-PUFA deficiency in meal-based diets. Moreover, because replacing fish meal alters dietary lipid supply, researchers have rarely distinguished the physiological effects of protein-source substitution from those of the concurrent change in dietary lipid.

Replacing fish meal with meat meal inevitably reduces the dietary supply of n-3 long-chain polyunsaturated fatty acids (LC-PUFA), raising the question of whether salmonids can maintain the n-3 LC-PUFA quality of their fillet either by conserving tissue LC-PUFA or by up-regulating endogenous biosynthesis. This question is nutritionally important because the EPA and DHA in fish muscle are a major dietary source of beneficial fatty acids for humans, and their reduction directly affects the nutritional value of the product. Therefore, the present study evaluated how graded replacement of fish meal with meat meal, with or without a concurrent reduction in dietary fish oil, affects the growth, muscle fatty acid composition, hepatic LC-PUFA metabolism, and related physiological responses of chum salmon.

## 2. Materials and Methods

### 2.1. Experimental Fish

Chum salmon maintained at the Aquafeed Research Center (Pohang, Republic of Korea) were used in the present study and acclimated to the experimental conditions for two weeks. The fish were juveniles of undetermined sex and were apparently healthy, showing no external signs of disease or abnormality. During acclimation, the fish were fed commercial sinking rainbow trout feed (Woosung Feed Co., Daejeon, Republic of Korea; 43% crude protein and 19% lipids). The fish were hand-fed to apparent satiation twice daily (09:00 and 15:30 h). During feeding, the behavior of the fish was closely observed, and feed was supplied gradually until they no longer accepted it, ensuring that little to no uneaten feed remained in the tank. After acclimation, fish with an average body weight of 173.3 ± 2.0 g were randomly assigned to dietary treatments in 1000 L circular fiberglass-reinforced plastic tanks in a flow-through system at a density of 15 fish per tank (3 tanks per dietary treatment). Feces and feed residues were removed daily via siphoning, and dead fish were removed immediately. The dissolved oxygen concentration was maintained with liquefied oxygen and vigorous aeration in each tank. The feed trial lasted 16 weeks; during this period, the mean water temperature, salinity, pH, and dissolved oxygen were 13.3 °C, 32.4 psu, 8.3, and 11.0 mg L^−1^, respectively, and a natural photoperiod was maintained. Fish were assigned to the dietary treatments in a completely randomized design, with the tanks of the different treatments interspersed across the flow-through system to minimize positional effects. All samples were labeled only with tank numbers, so that the analysts were unaware of the dietary treatment allocation during the analyses.

All procedures were performed in accordance with the ethical guidelines for experimental animals of the NIFS and approved by the Institutional Animal Care and Use Committee of the NIFS (Approval No. 2026-NIFS-IACUC-27).

### 2.2. Diet Preparation

A commercially available meat meal was used as the fish meal replacer. The control diet (D1) used 50% fish meal and 10% soybean meal as the main protein sources, fish oil as the lipid source, and wheat flour as the carbohydrate source (Table 1). In diets D2 and D3, the dietary fish meal was reduced from 50% to 35% and 20%, respectively, with the removed portion replaced by meat meal. To separate the effect of fish meal replacement from that of the dietary lipid level, diet D4 was formulated with the same fish meal and meat meal levels as those in D3 but with reduced fish oil. By adjusting the wheat flour and fish oil content, diets D1–D3 were formulated to be isonitrogenous and isolipidic (~49% crude protein and 20% crude lipid on a dry matter basis), whereas D4 contained the same protein but reduced lipid in accordance with its lower fish oil inclusion (Table 1). Crystalline L-lysine and DL-methionine were added to meat meal-based diets to match the essential amino acid profile of the control diet. Table 2 and Table 3 present the amino acid and fatty acid compositions of the experimental ingredients and diets, respectively. All ingredients were thoroughly mixed, and fish oil and water were added to form a dough, which was pelleted into 5 mm pellets using a pellet mill. The pellets were dried at 50 °C for 4 h and stored at 4 °C until use. Each experimental diet was assigned to triplicate groups.

### 2.3. Growth Performance and Biological Indices

At the end of the 16-week feeding trial, all fish were fasted for 24 h and anesthetized with 100 ppm phenoxyethanol. All surviving fish in each tank were counted and collectively weighed to determine survival rate and weight gain (WG). Four fish were then randomly selected from each tank, individually weighed, and measured for total length. The viscera and liver were removed to determine the viscerosomatic index (VSI) and hepatosomatic index (HSI).

Growth performance, feed utilization, and biological indices were calculated as follows:

Weight gain (WG, %) = (final body weight − initial body weight) × 100/initial body weight

Specific growth rate (SGR, %/day) = [(ln final weight − ln initial weight)] × 100/d reared]

Feed conversion ratio (FCR) = feed intake/wet WG

Daily feed intake (%, DFI) = (feed intake × 100)/[(initial body weight + final body weight + dead weight) × d/2]

Protein efficiency ratio (PER) = WG/protein intake

Condition factor (CF, g/cm^3^) = total body weight of fish (g) × 100/total length of fish (cm)^3^

VSI = viscera weight of fish × 100/total body weight of fish, and

HSI = liver weight of fish × 100/total body weight of fish

### 2.4. Sample Collection

After anesthetizing the fish at the end of the trial, blood was drawn from the caudal vasculature of three fish per tank (nine fish per dietary treatment) using 23-gauge needles (KOVAX-Syringe; Korean Vaccine, Seoul, Republic of Korea). A portion of the blood was collected from each fish using heparinized syringes for plasma, and the remainder using non-heparinized syringes for serum. Collected blood was immediately centrifuged at 2795× *g* for 10 min at 4 °C using a refrigerated centrifuge (VS-24SMTi; Vision S & Tech., Daejeon, Republic of Korea). Plasma and serum were stored at −80 °C until analysis. The livers of three fish per tank were excised, and a portion of each was immersed in RNAlater (Sigma-Aldrich, St. Louis, MO, USA) and stored at −80 °C for hepatic gene expression analysis. Dorsal muscle was collected from five fish per tank, pooled, and stored at −20 °C for proximate and fatty acid composition analysis.

### 2.5. Nutrient Composition

The proximate composition of the experimental ingredients, diets, and dorsal muscles was analyzed according to the standard methods of the Association of Official Analytical Chemists [19]. Crude protein (N × 6.25) was measured using an 8400 automatic Kjeldahl nitrogen determinator (FOSS, Hillerød, Denmark), and crude lipid was analyzed using a Soxtec System 2043 (FOSS). Moisture content was determined after oven-drying at 135 °C for 2 h, and ash content was quantified via incineration in a muffle furnace at 600 °C for 6 h. Total lipids were extracted using a chloroform/methanol mixture (2:1, *v*/*v*), as described previously [20]. Fatty acid methyl esters were prepared from the lipid extracts via methanolysis and analyzed using a gas chromatograph (Trace 1310, Thermo Scientific, Waltham, MA, USA) equipped with a flame ionization detector and an SP-2560 capillary column (100 m × 0.25 mm i.d., 0.2 μm film thickness; Supelco, Bellefonte, PA, USA). To analyze bound amino acid composition, add 20 mL of 6 N HCl to the sample, then vacuum-seal it and subject it to acid hydrolysis in a dry oven at 110 °C for 24 h. The hydrolysate was filtered through a glass filter, then the filtrate was vacuum-concentrated at 55 °C to evaporate the hydrochloric acid and water. The concentrate was adjusted to 25 mL with sodium citrate buffer (pH 2.20) in a volumetric flask, filtered through a 0.45 µm membrane filter, and analyzed using an automatic amino acid analyzer (Biochrom 30+; Biochrom Ltd., Cambridge, UK).

### 2.6. Blood Analysis

Plasma concentrations of glucose (Cat. No. 981780), total protein (Cat. No. 981827), triglycerides (Cat. No. 981786), alanine aminotransferase (ALT; Cat. No. 981759), and aspartate aminotransferase (AST; Cat. No. 981771) (all reagent kits from Thermo Fisher Scientific) were analyzed using a fully automatic wet chemistry analyzer (Indiko; Thermo Fisher Scientific), according to the manufacturer’s instructions.

Serum lysozyme, superoxide dismutase (SOD), glutathione peroxidase (GPx), catalase (CAT), and insulin-like growth factor-1 (IGF-1) were measured using commercial fish ELISA kits (CUSABIO Biotech Co., Ltd., Wuhan, China), according to the manufacturer’s instructions [SOD, Cat. No. CSB-E15929Fh; CAT, Cat. No. CSB-E15928Fh; GSH-PX (GPx), Cat. No. CSB-E15930Fh; lysozyme, Cat. No. CSB-E17296Fh; IGF-1, Cat. No. CSB-E12122Fh].

### 2.7. RNA Isolation

Total RNA was isolated from chum salmon liver tissue using RNAiso (Takara, Shiga, Japan) and purified with the yesR Total RNA plus kit (Genesgen, Busan, Republic of Korea), following the manufacturer’s instructions. Total RNA purity and concentration were measured using a BioTek Synergy HTX Multimode Microplate Reader (Agilent Technologies, Santa Clara, CA, USA). cDNA was synthesized for quantitative reverse transcription polymerase chain reaction (qRT-PCR) using the PrimeScript RT Master Mix (Takara). *EF1A* (elongation factor 1-alpha) was used as a reference gene to normalize concentrations across samples [21]. qRT-PCR was conducted on a QuantStudio 1 Real-Time PCR System (Applied Biosystems, Waltham, MA, USA) using PowerUp SYBR Green Master Mix (Applied Biosystems). Table 4 lists the primer sequences used for qRT-PCR analysis. Thermal cycling conditions were as follows: 50 °C for 2 min (one cycle) to activate uracil-DNA glycosylase, followed by 95 °C for 2 min (one cycle) to activate DNA polymerase. The main amplification step consisted of 45 cycles of denaturation at 95 °C for 15 s and annealing/extension at 60 °C for 20 s. To confirm reaction specificity, melt curve analysis was performed by heating at 95 °C for 15 s, then continuously increasing the temperature at 0.05 °C/s. The relative expression levels were calculated using the delta-delta Ct method [22] and are presented as the mean ± standard error (SE).

### 2.8. Statistical Analysis

All statistical analyses were performed using SPSS version 23 (IBM Corp., Armonk, NY, USA). Before analysis, the normality of data distribution and homogeneity of variances were examined using Shapiro–Wilk and Levene’s tests, respectively. The overall effect of dietary treatment was evaluated using a one-way analysis of variance (ANOVA), and significant differences among the four dietary groups were identified using Tukey’s honest significant difference (HSD) test for multiple pairwise comparisons among treatments. For the data that did not meet the assumptions, a Kruskal–Wallis test was used instead. The effect of reduced dietary fish oil under the same protein-source composition was assessed by comparing the D3 and D4 groups, which shared the same protein sources, using an independent *t*-test. All analyses used tank means (*n* = 3 tanks per treatment). Differences were considered significant at *p* < 0.05, and all data are presented as the mean ± SE.

## 3. Results

### 3.1. Growth Performance and Feed Utilization

Growth performance, feed utilization, and biological indices of chum salmon after the 16-week feeding trial are presented in Table 5. Replacing dietary fish meal with meat meal did not significantly affect survival. Final body weight ranged from 270.6 ± 14.1 g (D4) to 304.4 ± 23.8 g (D1), and WG ranged from 55.2 ± 8.09% to 74.7 ± 13.3%, respectively. Growth performance and feed utilization decreased with increasing meat-meal inclusion; however, the differences among treatments were not significant (*p* > 0.05). Dietary treatments did not significantly affect the CF or VSI (*p* > 0.05), whereas they significantly affected the HSI (*p* < 0.05), with fish fed D1 exhibiting a significantly higher value (1.10 ± 0.01%) than did those fed D3 (0.96 ± 0.01%) and D4 (0.95 ± 0.03%). HSI decreased progressively with increasing dietary meat-meal inclusion. Dietary lipid levels did not differ.

### 3.2. Proximate Composition of Dorsal Muscle

The proximate composition of the dorsal muscles is shown in Table 6. The experimental diets did not significantly affect muscle moisture, crude protein, or crude lipid contents (*p* > 0.05). Muscle ash content was significantly higher in fish fed D4 than in those fed D1 (*p* < 0.05). Muscle crude lipid content was significantly lower, and crude protein content significantly higher in fish fed D4 than in those fed D3 (*p* < 0.05). In contrast, muscle ash content did not differ significantly between the groups (*p* > 0.05).

### 3.3. Fatty Acid Composition of Dorsal Muscle

The fatty acid composition of the dorsal muscles is shown in Table 7. The experimental diets significantly affected most of the major fatty acids (*p* < 0.05); however, 16:1 and 20:2n-6 did not differ significantly among treatments (*p* > 0.05). The muscle contents of 16:0, 18:0, and 18:1n-9 increased significantly as dietary meat-meal inclusion increased, whereas those of 14:0, 20:1n-9, 22:1n-9, 20:5n-3 (eicosapentaenoic acid; EPA), and 22:6n-3 (docosahexaenoic acid; DHA) decreased significantly (*p* < 0.05). Accordingly, total n-3 fatty acids (Σn-3) decreased significantly with increasing meat-meal inclusion (*p* < 0.05). Evaluation of the effect of dietary fish oil level between D3 and D4 revealed that fish fed D4 exhibited significantly higher muscle 16:0, 18:0, 18:1n-9, 18:2n-6, and Σn-6, and significantly lower 14:0, 20:1n-9, and 22:1n-9 than did those fed D3 (*p* < 0.05). Muscle EPA was significantly lower, and DHA significantly higher in fish fed D4 than in those fed D3 (*p* < 0.05); however, Σn-3 did not differ significantly between the groups (*p* > 0.05).

### 3.4. Plasma and Serum Parameters

Plasma biochemical parameters are presented in Table 8. The experimental diets did not significantly affect plasma ALT, AST, glucose, total protein, and triglyceride levels (*p* > 0.05). No significant differences were observed between D3 and D4 for any plasma parameters (*p* > 0.05).

### 3.5. Serum Immune, Antioxidant, and Endocrine Parameters

Serum non-specific immune and antioxidant parameters are shown in Table 9. The experimental diets did not significantly affect serum lysozyme, SOD, and IGF-1 levels (*p* > 0.05). In contrast, dietary treatments significantly affected serum GPx and catalase activities (*p* < 0.05). Both enzyme activities were the lowest in fish fed D3, and both were significantly higher in fish fed D4 than in those fed D3 (*p* < 0.05).

### 3.6. Hepatic Gene Expression

Figure 1 shows the relative hepatic expression levels of the target genes. Among the lipid and LC-PUFA metabolism genes, *srebf1* (sterol regulatory element-binding transcription factor 1, a master regulator of lipogenesis and fatty acid biosynthesis) was significantly affected by the overall dietary treatment (*p* = 0.024), being the highest in fish fed D3 and significantly higher than those in fish fed D1 and D2. *elovl5* (elongation of very long-chain fatty acids protein 5, a fatty acid elongase) expression was significantly lower in fish fed D4 than in those fed D3 (D3 vs. D4, *p* = 0.037), and *fads2* (fatty acid desaturase 2, which introduces double bonds into fatty acid chains) and *pparα* (peroxisome proliferator-activated receptor α, a regulator of fatty acid oxidation) expression were also lower in D4, although these differences were not significant. The taurine biosynthesis genes *cdo1* (cysteine dioxygenase 1) and *csad* (cysteine sulfinic acid decarboxylase), and the immune-related genes *nfkb1*, *nfkb2*, and *il1β* (interleukin-1β), were not significantly affected by the dietary treatment (*p* > 0.05). However, *cdo1* showed a tendency toward lower expression in fish fed D4 (D3 vs. D4, *p* = 0.055).

## 4. Discussion

In this study, dietary treatment did not significantly affect growth performance or feed utilization, indicating that meat meal could replace up to 60% of dietary fish meal in chum salmon without impairing growth. Although final body weight, WG, and SGR decreased with increasing meat-meal inclusion, the differences were not significant. This finding indicates that meat meal can serve as a suitable protein source for inclusion at high replacement levels in low-fish-meal diets for chum salmon.

Animal protein sources have a more balanced nutritional profile than plant protein sources [23,24]. The meat meal used in this study was prepared from meat by-products without bones and had a high crude protein content because of its protein-rich raw materials. This type of meat meal shows pepsin digestibility comparable to fish meal and soy protein concentrate [5]. However, unlike the present study, Lee and Lee [25] showed that dietary fish meal could be replaced with up to 29.1% meat meal when juvenile rockfish were fed diets with 14.5, 29.1, 43.6, or 58.2% meat meal for eight weeks. However, WG and feed efficiency decreased as replacement levels increased. This difference suggests that the replacement level of fish meal depends largely on meat meal quality, and the higher replacement level achieved in this study likely reflects the favorable protein quality of the meat meal used, along with supplementation of limiting amino acids such as methionine. Fortifying ingredients that are deficient or low in certain amino acids essential for growth can improve overall diet quality [6,16,26,27]. The palatability of low-fish-meal diets is another common concern, as replacing fish meal with animal by-products reduces feed intake and, consequently, fish growth in several species [6,28,29,30]. Adding feed attractants to low-fish-meal diets may improve palatability and feed consumption [31,32]. However, in the present study, daily feed intake did not differ significantly among treatments, indicating that including meat meal did not reduce diet palatability over the feeding period and that feed attractants were not required at the tested replacement levels.

Consistent with the growth results, dietary treatments did not affect VSI. However, HSI decreased significantly with increasing meat-meal inclusion. HSI did not differ between the two diets containing the same protein sources but different fish oil levels, indicating that the reduction was related to fish meal replacement rather than dietary lipid levels. The response of HSI to fish-meal replacement varies among species. A low HSI, which may indicate hepatic atrophy or altered hepatic allocation of metabolic energy, has been observed in catfish fed poultry by-product meal [33] and in rainbow trout fed diets high in plant protein sources, suggesting that HSI reflects changes in hepatic metabolic activity or energy partitioning [34]. In contrast, replacing high levels of fish meal with animal protein blends increased the HSI and induced hepatic steatosis in hybrid groupers and Japanese sea bream [26,35]. In this study, HSI decreased with meat-meal inclusion; however, plasma ALT and AST activities, markers of hepatic damage, were not affected by the dietary treatments. This finding indicates that the lower HSI in fish fed meat-meal-based diets may reflect reduced hepatic lipid accumulation rather than hepatic steatosis or liver dysfunction. However, since hepatic lipid content and liver histology were not directly evaluated, this inference warrants further histological confirmation. It further suggests that the liver response to fish meal replacement is species- and ingredient-dependent and that the meat meal used in the present study did not adversely affect hepatic health.

When up to 60% of fish meal protein was replaced with meat meal (D1–D3), moisture, crude protein, or crude lipid contents of the fish dorsal muscle did not significantly differ. This result suggests that meat meal is a high-quality protein source that can replace fish meal without negatively affecting muscle composition and nutrient accumulation in the target species. These results are consistent with those of previous studies reporting that dietary inclusion of meat meal does not affect the body composition of rockfish [17] or red sea bream [36]. In contrast, under the same meat-meal replacement conditions, the fish fed D4 exhibited a significant decrease in muscle crude lipid content and a significant increase in moisture and crude protein content compared with those fed D3. Similarly, muscle lipid content was significantly higher in rainbow trout in a high dietary lipid group (30%) than in a low dietary lipid group (15%) [37]. Furthermore, several previous studies have confirmed a tendency toward decreased muscle protein content in fish fed high-lipid diets [38,39,40,41,42], aligning with the significant increase in crude protein content observed in the present study when dietary lipid energy was restricted (D4).

The fatty acid composition of the dorsal muscle largely resembled that of the experimental diets, which agrees with the observation that the fatty acid composition of fish fillets reflects the dietary fatty acid composition [43]. When dietary fish meal was replaced with meat meal, the muscle contents of 18:0 and 18:1n-9 increased, whereas those of 20:1n-9, 22:1n-9, EPA, and DHA decreased. This finding is consistent with the fatty acid characteristics of meat meal, which is rich in saturated and monounsaturated fatty acids but largely devoid of n-3 LC-PUFA. Consequently, the total muscle n-3 PUFA content decreased with increasing meat meal intake. Gunathilaka et al. [9] also reported a reduction in muscle n-3 PUFA following fish meal replacement in chum salmon, and a comparable decrease in n-3 LC-PUFA was observed in the whole body of red sea bream with increasing meat meal inclusion [36]. These reports support the notion that the deposition of n-3 LC-PUFA in fish muscle is highly dependent on its dietary supply. Indeed, between D3 and D4, muscle EPA decreased significantly whereas DHA increased significantly, although total n-3 fatty acids (Σn-3) did not differ. This indicates a shift in the relative partitioning between EPA and DHA while the total n-3 content was maintained. This pattern may be partly explained by the preferential retention of DHA in membrane phospholipids and by a compositional effect of the lower muscle lipid content in D4, in which a smaller neutral-lipid fraction can increase the relative proportion of structural fatty acids such as DHA.

In this study, total muscle n-3 PUFA content did not differ between D3 and D4 diets. This suggests that fish meal replacement was the primary factor associated with the decline in muscle n-3 PUFA. However, because reducing the dietary fish oil level was accompanied by changes in wheat flour inclusion, total dietary lipid, and the overall composition, the D3–D4 comparison does not fully isolate the effect of dietary fish oil, and this interpretation should be treated with caution. This reduction in muscle n-3 LC-PUFA represents the principal trade-off of replacing fish oil, as the EPA and DHA contents of the fillet largely determine its nutritional value for human consumption [44,45,46]. From a nutritional perspective, omega-3 long-chain polyunsaturated fatty acids (n-3 LC-PUFAs), such as EPA and DHA, offer diverse health benefits. As essential fatty acids that cannot be synthesized in vivo, they must be acquired through the diet. They have been reported to reduce the incidence of cardiovascular diseases by improving serum triglyceride levels and blood circulation [47]. In particular, they are key nutrients that provide substantial benefits in alleviating hyperlipidemia and improving liver function. Although the recommended daily intake varies slightly among national health authorities, the general consensus recommends consuming fish at least twice a week, including at least one serving of oily fish [48]. Given that farmed fish production is vital to meeting this nutritional demand, our findings suggest that despite the dietary fish meal replacement, the salmon retain sufficient nutritional value to supply the EPA and DHA required for human health.

Most plasma biochemical parameters, including glucose, total protein, and triglyceride levels, were unaffected by the dietary treatments, indicating that replacement of fish meal with meat meal did not induce metabolic disturbances in chum salmon. ALT and AST leak from damaged cells into the bloodstream under hepatic injury conditions and are therefore widely used as corresponding biomarkers, with ALT being the more liver-specific of the two. Both ALT and AST were unaffected across all treatments, supporting the view that including meat meal did not impair hepatic function, even at the highest replacement level [49,50,51,52].

Changes in muscle fatty acid composition are closely associated with the hepatic expression of lipid- and LC-PUFA metabolism-related genes. The expression of *srebf1*, a master transcriptional regulator of lipogenesis and fatty acid biosynthesis, was the highest in fish fed D3, the diet with the highest meat meal inclusion. In salmonids, the LC-PUFA biosynthetic pathway is regulated through the *Lxr*–*Srebp-1* axis, with high dietary levels of EPA and DHA suppressing the pathway, whereas low supply de-represses it [18,53,54,55,56]. Therefore, elevated *srebf1* expression in fish fed the D3 diet may represent an initial transcriptional response to the reduced dietary supply of n-3 LC-PUFA associated with fish meal replacement.

Under normal conditions, SREBP-1 activates the transcription of the downstream biosynthetic genes *fads2* and *elovl5* to promote endogenous LC-PUFA synthesis [18]. However, this transcriptional response did not translate into upregulation of *fads2* and *elovl5*; they were not induced in fish fed D3, and *elovl5* was significantly downregulated in fish fed D4. A likely reason for this is the limited availability of biosynthetic substrates in meat-meal-based diets. Endogenous EPA and DHA synthesis proceeds from the C18 precursors 18:3n-3 and 18:2n-6 through the sequential action of Fads and Elovl enzymes [18,53,54]. In salmonids, this pathway is typically induced when fish oil is replaced with plant oils that are rich in these precursors [57]. In contrast, meat meal is poor not only in n-3 LC-PUFA but also in their C18 precursors. Consequently, even though the reduced dietary EPA and DHA may have relieved *srebf1* suppression, the scarcity of C18 substrates in meat-meal-based diets would have constrained the endogenous synthesis of n-3 LC-PUFA, such that elevated *srebf1* expression was not accompanied by activation of the downstream pathway.

Salmonids underwent a specific fourth round of whole-genome duplication (Ss4R), which increased genome complexity and generated duplicated genes (paralogs) [58,59]. For instance, unlike most teleosts where *elovl5* is kept as a singleton, salmonids possess two duplicated *elovl5* genes that are subfunctionalized with distinct spatial expression patterns in the liver and intestine [60,61]. Given this genomic complexity, it is highly plausible that these specific paralogs are differentially regulated by nutritional stimuli and *SREBP-1*. Therefore, the lack of transcriptional induction observed in our study might be restricted to the specific *fads2* and *elovl5* isoforms analyzed.

The dietary treatments did not affect serum immune, antioxidant, and endocrine parameters, including serum lysozyme, and IGF-1 levels or the hepatic expression of the immune-related genes *nfkb1*, *nfkb2*, and *il1β*. This finding indicates that replacing fish meal with meat meal, even at the highest inclusion level, did not provoke a hepatic inflammatory response or compromise the non-specific immunity of chum salmon. In contrast, the serum activities of the antioxidant enzymes GPx and catalase were the lowest in fish fed D3 and higher in the other groups, suggesting that the antioxidant status was altered at the highest level of meat meal inclusion. However, because oxidative stress markers, such as lipid peroxidation, were not measured and variation among replicate groups was relatively large, the physiological significance of this response remains unclear and warrants further investigation.

## 5. Conclusions

Meat meal is a promising and practical alternative protein source for chum salmon diets, provided that limiting amino acids are appropriately balanced. In the present study, replacing up to 60% of dietary fish meal with meat meal did not significantly affect growth performance or feed utilization, although some growth parameters decreased numerically with increasing meat meal inclusion; given the limited number of replicate tanks, this finding should be interpreted with some caution. The dietary lipid level could also be reduced without a significant loss of growth, which may be advantageous from a production standpoint. However, high fish meal replacement reduced the n-3 LC-PUFA content of the fillet, representing a key trade-off for its nutritional quality. Building on these findings, future research should explore the supplementation of meat meal-based diets with concentrated sources of n-3 LC-PUFA, such as DHA-rich microalgal (*Schizochytrium* sp.) powders or oils, to offset this decline and improve the nutritional quality of the product. Further studies are warranted to develop practical diets optimized for different growth stages of chum salmon, thereby providing a basis for producing high-quality fish that meet the nutritional expectations of consumers.

## Figures and Tables

**Figure 1:**
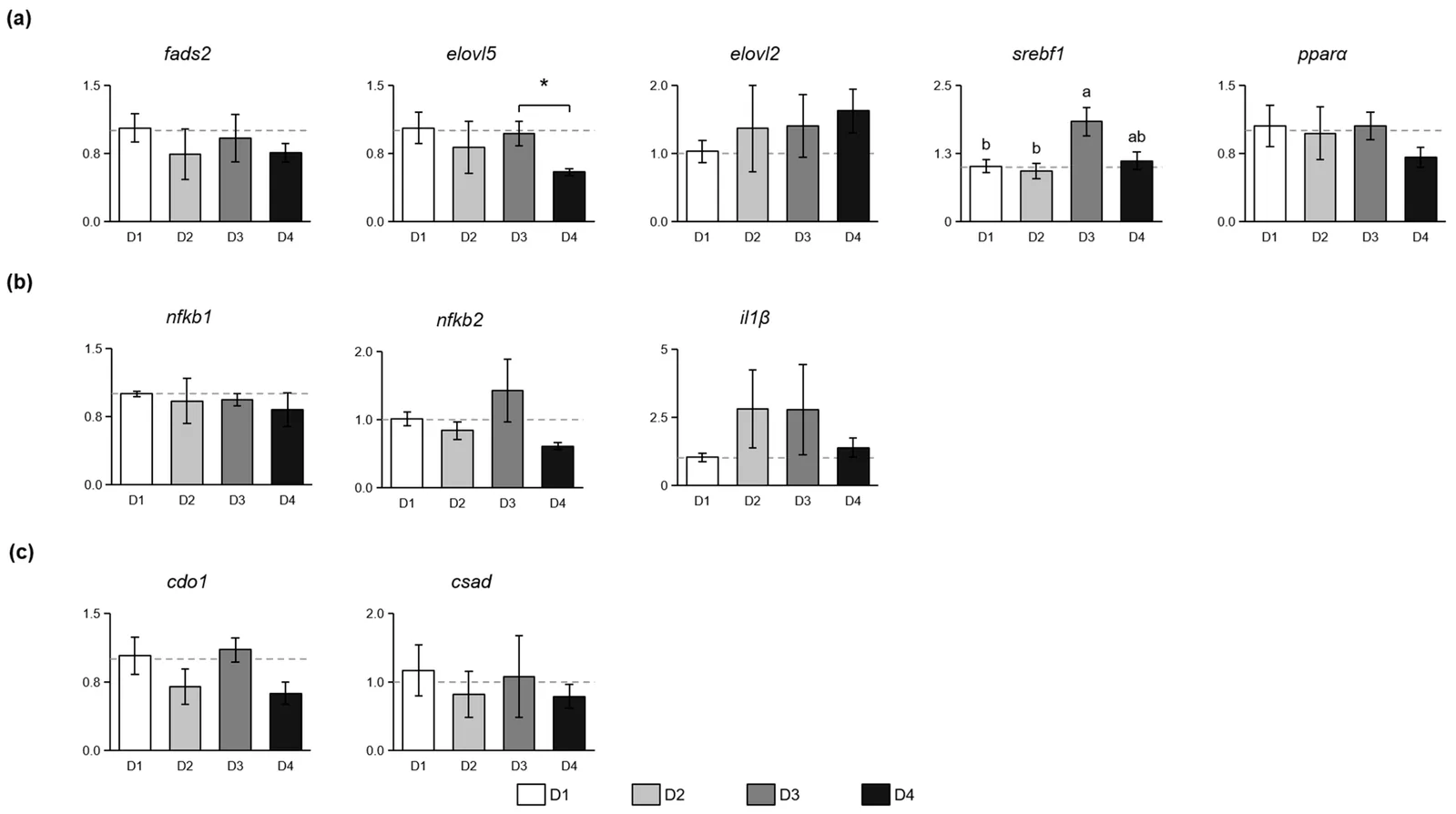
Relative hepatic mRNA expression of genes related to (**a**) lipid and n-3 long-chain polyunsaturated fatty acid (LC-PUFA) metabolism, (**b**) immune response, and (**c**) taurine metabolism in chum salmon fed the four experimental diets. Values are means ± standard error (*n* = 3), expressed as fold change relative to the control diet D1 (2^−ΔΔCt^ method, normalized to *ef1a*). The dashed line indicates the control level. Different letters above bars denote significant differences among treatments (Tukey’s test, *p* < 0.05); * denotes a significant difference between D3 and D4 (*t*-test, *p* < 0.05).

**Table 1:** Ingredients (g 100 g^−1^ diet) and proximate composition (%) of four experimental diets.

	Experimental Diets			
	D1	D2	D3	D4
Ingredient composition (g 100 g^−1^ diet)				
Fish meal ᵃ	50.0	35.0	20.0	20.0
Meat meal ᵇ	–	17.0	34.0	34.0
Soybean meal	10.0	10.0	10.0	10.0
Wheat gluten	3.0	3.0	3.0	3.0
Wheat flour	18.0	16.8	15.6	20.7
Fish oil	15.0	13.6	12.2	7.1
Monocalcium phosphate	1.0	1.0	1.0	1.0
Vitamin premix	1.0	1.0	1.0	1.0
Mineral premix	1.0	1.0	1.0	1.0
Lysine	–	0.3	0.6	0.6
Methionine	–	0.3	0.6	0.6
Choline chloride	0.5	0.5	0.5	0.5
Stay C	0.5	0.5	0.5	0.5
Proximate composition (% of dry matter)				
Dry matter	96.6	96.5	96.2	96.1
Crude protein	48.8	49.5	49.1	51.1
Crude lipid	20.4	20.9	21.4	15.2
Ash	11.1	11.7	12.0	12.3

D1, control diet containing 50% fish meal without meat meal; D2, 30% of the fish meal of D1 replaced by meat meal; D3, 60% of the fish meal of D1 replaced by meat meal; D4, same protein source combination as D3 but with reduced dietary fish oil. ᵃ Fish meal (Blumar, Santiago, Chile; 69.6% crude protein, 8.0% crude lipid, and 13.3% ash on an as-fed basis). ᵇ Meat meal (The Feed, Seoul, Republic of Korea; 63.6% crude protein, 15.4% crude lipid, and 17.0% ash on an as-fed basis). –, not included.

**Table 2:** Essential amino acid composition of protein sources and four experimental diets (% in protein).

	Protein Source		Experimental Diet			
	Fish Meal	Meat Meal	D1	D2	D3	D4
Arginine	6.23	6.85	6.12	6.02	6.37	6.06
Histidine	3.21	2.66	2.04	2.08	2.07	2.05
Isoleucine	4.34	2.76	3.79	3.88	3.71	3.80
Leucine	6.94	5.13	6.80	6.99	6.59	6.83
Lysine	7.25	5.47	7.85	7.99	7.57	7.86
Methionine	2.10	0.88	2.41	2.40	2.53	2.33
Phenylalanine	4.32	3.17	4.08	4.09	4.15	4.08
Threonine	4.78	3.35	4.25	4.30	4.14	4.23
Valine	5.52	4.57	4.56	4.57	4.42	4.50
ΣEAA	44.69	34.84	41.90	42.32	41.55	41.74

ΣEAA, sum of essential amino acids.

**Table 3:** Fatty acid composition (% of total fatty acids) of protein sources and four experimental diets.

	Protein Source		Experimental Diet			
	Fish Meal	Meat Meal	D1	D2	D3	D4
14:0	7.31	3.27	6.93	6.42	6.08	5.58
16:0	28.3	30.7	14.2	16.0	19.0	21.1
16:1	7.39	3.93	5.88	5.66	5.57	5.31
18:0	5.27	19.2	3.54	5.31	7.45	8.49
18:1n-9	14.8	42.9	14.5	18.4	20.9	23.7
18:2n-6	7.07	n.d.	3.10	2.95	3.61	4.76
20:1n-9	n.d.	n.d.	17.1	15.7	14.1	11.5
20:2n-6	0.38	n.d.	2.48	2.11	n.d.	n.d.
22:1n-9	n.d.	n.d.	12.4	11.3	10.1	8.07
20:5n-3 (EPA)	16.0	n.d.	12.0	10.1	8.39	7.34
22:6n-3 (DHA)	13.5	n.d.	7.85	6.07	4.72	4.06
ΣSFA	40.9	53.2	24.7	27.7	32.6	35.2
ΣMUFA	22.2	46.8	49.9	51.1	50.7	48.6
ΣPUFA	37.0	n.d.	25.4	21.2	16.7	16.2
Σn-3	29.5	n.d.	19.8	16.1	13.1	11.4
Σn-6	7.45	n.d.	5.57	5.06	3.61	4.76

ΣSFA, total saturated fatty acids; ΣMUFA, total monounsaturated fatty acids; ΣPUFA, total polyunsaturated fatty acids; EPA, eicosapentaenoic acid; DHA, docosahexaenoic acid; n.d., not detected. D1, control diet containing 50% fish meal protein without meat meal; D2, 30% of the fish meal protein of D1 replaced by meat meal; D3, 60% of the fish meal protein of D1 replaced by meat meal; D4, same protein source combination as D3, but with reduced dietary fish oil.

**Table 4:** Primer sequences used for quantitative real-time PCR analysis of chum salmon.

Gene	GenBank Accession No.	Forward Primer (5′–3′)	Reverse Primer (5′–3′)
*nfkb1*	XM_035744233.2	TCTGGAGGAAAGGTCAAGGA	ATCCGTCCAGCTTCAGATCA
*nfkb2*	XM_052498463.1	ACAGCATGGACGCAGAAAGT	AGCAGTTTGTAGCAGGGAGA
*il1b*	XM_035786315.2	GGCATAAAGGGATCCAACCT	CGGTGTTCCGTCTGTAGAAA
*fads2*	XM_052481012.1	GGTTCGTGCTTTGTGTGAGA	AGGTCGTTAATCTGCGATGG
*elovl5*	XM_035800980.2	CAGAAGCTGGCTGTTTTTCC	CCGCACTTTCCTGTGTGTAA
*elovl2*	XM_052483872.1	ATGCCTGCTCTTCCAGTTCT	TGTCTTGACAACCAGGTCCA
*srebf1*	XM_052513476.1	TGCAGCTGACGAACAGACAT	TGAAGTCCTCCCCTGCTAAA
*pparα*	XM_035737699.2	TGGAACCCAAGTTCCAGTTC	AAGGTGTCGTCTGGGTGATT
*cdo1*	XM_035784857.2	AAGGTTCGGTCAGTTTGCAC	CTGCGCCTCAGTTGTTTTCT
*csad*	XM_035798953.1	CAAAGGTGGCTCCAGTGATT	GATCATTCCCAAGCCTCTCA
*ef1a*	MK989711.1	AGCTCAAGGAGAAGATCGAC	CTTGATGACACCAACAGCCA

**Table 5:** Growth performance and feed utilization of fish fed different experimental diets.

Parameter	Experimental Diet				*p*-Value	
	D1	D2	D3	D4	ALL	D3 vs. D4
IAW (g)	174.2 ± 0.61	172.6 ± 0.11	172.2 ± 0.09	174.3 ± 2.30	0.542	0.465
FBW (g)	304.4 ± 23.8	292.6 ± 26.6	285.1 ± 10.3	270.6 ± 14.1	0.688	0.452
WG (%)	74.7 ± 13.3	69.5 ± 15.4	65.6 ± 6.09	55.2 ± 8.09	0.677	0.365
SGR (%/day)	0.49 ± 0.07	0.46 ± 0.08	0.45 ± 0.03	0.39 ± 0.05	0.689	0.360
DFI (%)	0.83 ± 0.03	0.81 ± 0.07	0.79 ± 0.03	0.77 ± 0.03	0.778	0.629
FCR	1.77 ± 0.17	1.92 ± 0.27	1.83 ± 0.07	2.04 ± 0.14	0.733	0.253
PER	1.18 ± 0.11	1.09 ± 0.14	1.12 ± 0.04	0.97 ± 0.07	0.542	0.146
CF (g/cm^3^)	0.92 ± 0.01	0.92 ± 0.02	0.95 ± 0.00	0.94 ± 0.02	0.543	0.638
HSI (%)	1.10 ± 0.01 ^a^	1.03 ± 0.05 ^ab^	0.96 ± 0.01 ^b^	0.95 ± 0.03 ^b^	0.029	0.772
VSI (%)	5.48 ± 0.05	5.43 ± 0.16	5.46 ± 0.26	5.84 ± 0.14	0.350	0.274
Survival (%)	100.0 ± 0.0	97.8 ± 2.2	93.3 ± 3.8	100.0 ± 0.0	0.193	0.158

Values (means ± SE) with various superscript lowercase letters within a row indicate statistically significant differences (*p* < 0.05), unlike mean values without superscripts. IAW, initial average weight; FBW, final body weight; WG, weight gain; SGR, specific growth rate; DFI, daily feed intake; FCR, feed conversion ratio; PER, protein efficiency ratio. CF, condition factor; HSI, hepatosomatic index; VSI, viscerosomatic index.

**Table 6:** Proximate composition (% wet weight) of dorsal muscle of fish fed different experimental diets.

	Experimental Diet				*p*-Value	
	D1	D2	D3	D4	ALL	D3 vs. D4
Moisture	65.3 ± 0.71	66.5 ± 0.75	65.2 ± 0.34	67.0 ± 0.04	0.125	0.007
Crude protein	19.5 ± 0.12	19.5 ± 0.04	19.3 ± 0.05	19.7 ± 0.10	0.084	0.030
Crude lipid	13.2 ± 0.78	13.2 ± 0.53	14.2 ± 0.26	12.0 ± 0.15	0.077	0.002
Ash	1.76 ± 0.02 ^b^	1.81 ± 0.01 ^ab^	1.79 ± 0.04 ^ab^	1.88 ± 0.02 ^a^	0.045	0.118

Values (means ± SE) with various superscript lowercase letters within a row indicate statistically significant differences (*p* < 0.05), unlike mean values without superscripts.

**Table 7:** Major fatty acid composition (% of total fatty acids) of dorsal muscle of fish fed different experimental diets.

	Experimental Diet				*p*-Value	
	D1	D2	D3	D4	ALL	D3 vs. D4
14:0	4.72 ± 0.04 ^a^	4.40 ± 0.06 ^b^	4.13 ± 0.09 ^b^	3.77 ± 0.04 ^c^	<0.001	0.022
16:0	16.5 ± 0.18 ^b^	17.1 ± 0.15 ^b^	17.3 ± 0.24 ^b^	18.8 ± 0.11 ^a^	<0.001	0.004
16:1	4.86 ± 0.06	4.69 ± 0.02	4.65 ± 0.06	4.63 ± 0.05	0.051	0.759
18:0	4.25 ± 0.06 ^d^	5.03 ± 0.01 ^c^	5.43 ± 0.08 ^b^	5.84 ± 0.05 ^a^	<0.001	0.012
18:1n-9	22.2 ± 0.50 ^d^	25.0 ± 0.28 ^c^	26.9 ± 0.20 ^b^	28.8 ± 0.38 ^a^	<0.001	0.012
18:2n-6	7.75 ± 0.11 ^b^	7.81 ± 0.43 ^b^	7.44 ± 0.21 ^b^	8.97 ± 0.09 ^a^	0.011	0.002
20:1n-9	12.4 ± 0.33 ^a^	11.3 ± 0.41 ^ab^	10.3 ± 0.20 ^b^	8.27 ± 0.08 ^c^	<0.001	0.001
20:2n-6	2.00 ± 0.15	1.93 ± 0.05	1.84 ± 0.10	1.74 ± 0.04	0.334	0.441
22:1n-9	7.32 ± 0.15 ^a^	6.45 ± 0.32 ^b^	5.79 ± 0.09 ^b^	4.47 ± 0.08 ^c^	<0.001	<0.001
20:5n-3 (EPA)	6.54 ± 0.25 ^a^	5.82 ± 0.06 ^b^	5.05 ± 0.12 ^c^	4.43 ± 0.10 ^c^	<0.001	0.016
22:6n-3 (DHA)	9.47 ± 0.24 ^a^	8.94 ± 0.28 ^a^	7.50 ± 0.08 ^b^	8.01 ± 0.08 ^b^	<0.001	0.012
Σn-3	16.0 ± 0.34 ^a^	14.8 ± 0.22 ^b^	12.5 ± 0.19 ^c^	12.4 ± 0.15 ^c^	<0.001	0.667
Σn-6	7.75 ± 0.11 ^ab^	7.81 ± 0.43 ^ab^	7.44 ± 0.21 ^b^	8.97 ± 0.09 ^a^	0.027	0.002

Values (means ± SE) with various superscript lowercase letters within a row indicate statistically significant differences (*p* < 0.05), unlike mean values without superscripts. EPA, eicosapentaenoic acid; DHA, docosahexaenoic acid.

**Table 8:** Plasma biochemical parameters of fish fed different experimental diets.

	Experimental Diet				*p*-Value	
	D1	D2	D3	D4	ALL	D3 vs. D4
ALT (U L^−1^)	6.43 ± 0.92	7.81 ± 0.63	6.80 ± 0.93	7.98 ± 1.34	0.638	0.508
AST (U L^−1^)	254 ± 38	206 ± 78	299 ± 2	211 ± 89	0.668	0.429
Glucose (mg dL^−1^)	101 ± 5	98.5 ± 10.8	96.5 ± 8.3	99.6 ± 8.2	0.982	0.806
Total protein (g dL^−1^)	3.93 ± 0.11	3.77 ± 0.23	3.90 ± 0.04	3.59 ± 0.21	0.498	0.216
Triglyceride (mg dL^−1^)	151 ± 25	146 ± 11	193 ± 20	133 ± 5	0.164	0.093

Values are means of triplicate groups and are presented as means ± SE. The absence of superscript letters indicates no significant differences among treatments. ALT, alanine aminotransferase; AST, aspartate aminotransferase.

**Table 9:** Serum immune, antioxidant, and endocrine parameters of fish fed different experimental diets.

Parameter	Experimental Diet				*p*-Value	
	D1	D2	D3	D4	ALL	D3 vs. D4
Lysozyme (ng mL^−1^)	14.4 ± 1.20	14.1 ± 1.63	10.3 ± 2.44	11.5 ± 1.36	0.314	0.684
SOD (ng mL^−1^)	3.12 ± 0.93	2.87 ± 0.06	5.09 ± 2.06	4.43 ± 0.67	0.526	0.778
GPx (μU mL^−1^)	2954 ± 140 ^a^	3981 ± 259 ^a^	1267 ± 460 ^b^	3536 ± 411 ^a^	0.002	0.021
Catalase (mU ml ^−1^)	295 ± 14 ^a^	192 ± 19 ^ab^	110 ± 11 ^b^	275 ± 1 ^ab^	0.024	<0.001
IGF-1 (pg mL^−1^)	3178 ± 189	3400 ± 264	3821 ± 92	3404 ± 215	0.226	0.149

Values (means ± SE) with various superscript lowercase letters within a row indicate statistically significant differences (*p* < 0.05), unlike mean values without superscripts. SOD, superoxide dismutase; GPx, glutathione peroxidase; IGF-1, insulin-like growth factor-1.

## Data Availability

The data presented in this study are available on request from the corresponding authors. The data are not publicly available due to institutional data management policy of the National Institute of Fisheries Science.

## Abbreviations

The following abbreviations are used in this manuscript:

Σn-3	Total n-3 fatty acids
ALT	Alanine aminotransferase
ANOVA	Analysis of variance
AST	Aspartate aminotransferase
CF	Condition factor
DFI	Daily feed intake
DHA	Docosahexaenoic acid
Elovl	Elongases of very long-chain fatty acids
EPA	Eicosapentaenoic acid
Fads	Fatty acid desaturases
FCR	Feed conversion ratio
GPx	Glutathione peroxidase
HSI	Hepatosomatic index
IGF-1	Insulin-like growth factor-1
LC-PUFA	n-3 long-chain polyunsaturated fatty acid
PER	Protein efficiency ratio
SE	Standard error
SGR	Sspecific growth rate
SOD	Ssuperoxide dismutase
VSI	Vviscerosomatic index
WG	Wweight gain
